# Microplastic Contamination in Amphibians and Reptiles: An Ecotoxicological Synthesis of Exposure, Mechanisms, and Risk Implications

**DOI:** 10.3390/toxics14060522

**Published:** 2026-06-15

**Authors:** Ahmet Ali Berber, Cansu Akbulut, Şefika Nur Demir, Muammer Kurnaz

**Affiliations:** 1 Vocational School of Health Services, Çanakkale Onsekiz Mart University, 17100 Canakkale, Türkiye; aberber@comu.edu.tr; 2 Department of Biology, Faculty of Science, Sakarya University, 54100 Sakarya, Türkiye; 3 Department of Molecular Biology and Genetics, Faculty of Science, Ataturk University, 25240 Erzurum, Türkiye; sefikademir@atauni.edu.tr; 4 Kelkit Sema Doğan Vocational School of Health Services, Gümüşhane Üniversitesi, 29600 Gümüşhane, Türkiye; muammerkurnazz@gmail.com

**Keywords:** microplastic, nanoplastic, herpetofauna, amphibia, Reptilia, bioaccumulation, ecotoxicology, ecological risk assessment, trophic transfer, endocrine disruption, oxidative stress, polymer additives, Trojan horse mechanism

## Abstract

Microplastic (MP) contamination has become a defining feature of twenty-first century environmental change, yet the toxicological and ecological consequences for amphibians and reptiles—two vertebrate classes already facing severe extinction pressures—remain fragmented across taxa, regions, and methodological traditions. Here, we synthesize field and experimental evidence from five continents to provide a taxonomically balanced, mechanistically grounded, and geographically explicit assessment of MP exposure, bioaccumulation, and toxicity in herpetofauna, drawing on a structured literature search in Web of Science, Scopus, and PubMed (January 2015—March 2026). Field detection rates of MPs in amphibian larvae range from 26% in conservatively screened Central European populations to 73–80% in anuran tadpoles from high-anthropogenic-pressure Anatolian catchments, with fibrous polyethylene terephthalate (PET), polyethylene (PE), and polypropylene (PP) particles dominating the detected burden. Mechanistic evidence converges on oxidative stress cascades, hypothalamic–pituitary–thyroid axis disruption, gut and cutaneous microbiome dysbiosis, and compromised antiviral and antifungal immunity, with the latter potentially amplifying vulnerability to *Batrachochytrium dendrobatidis* and to ranavirus. Among reptiles, sea turtles display near-universal MP ingestion with documented maternal transfer to eggs; freshwater turtles, terrestrial squamates, and crocodilians remain critically understudied. Three structural asymmetries constrain current ecotoxicological risk characterization: taxonomic bias toward anurans and sea turtles, geographic bias toward the Global North, and experimental bias toward acute, supra-environmental laboratory exposures using pristine, single-polymer particles that fail to capture the chemical complexity of weathered field mixtures. We argue that MP burden may warrant consideration as a candidate stressor criterion within IUCN Red List assessments and within environmental risk assessment frameworks for freshwater and terrestrial biodiversity once a robust quantitative relationship between MP burden and demographic decline or population-level fitness has been established, and propose six hypothesis-driven research priorities: methodological standardization, reptile toxicokinetics, transgenerational epigenetics, MP–pathogen microbiome interactions and their translation into population viability models, temperature × MP interaction under climate warming, and population-genetic consequences of contemporary MP-driven selection, as the most tractable avenues for ecotoxicological progress and for the development of herpetofauna-specific risk characterization frameworks.

## Simple Summary

Microplastics, tiny plastic particles smaller than 5 mm, now contaminate fresh water, soil, and even remote mountain springs across the globe. This review brings together the available evidence on how these particles affect frogs, salamanders, caecilians, lizards, snakes, and turtles—animals that are already declining rapidly worldwide. We show that microplastics have been detected in every major amphibian and reptilian group studied so far, that they can reach sea turtle embryos before hatching, and that they appear to weaken these animals’ resistance to deadly fungal and viral diseases. Most existing studies focus on frogs and sea turtles in wealthy regions, leaving salamanders, caecilians, snakes, lizards, and crocodiles poorly understood. We argue that microplastic pollution may warrant consideration as a potential threat in conservation assessments and identify the most urgent next steps for research aimed at protecting these animals.

## 1. Introduction

Plastic has become the defining synthetic material of the modern era. Annual global plastic production now exceeds 400 million tons, and recent estimates place riverine and coastal discharges of plastic waste to the oceans at between 4.8 and 12.7 million tons per year [1,2]. Through ultraviolet weathering, mechanical abrasion, and microbial action, macroplastic debris progressively fragments into microplastics (MPs), synthetic polymer particles smaller than 5 mm, and ultimately into nanoplastics (NPs) below 1 µm [3,4]. In addition to these secondary particles, MPs also enter the environment directly as primary microplastics manufactured at microscale (microbeads, pre-production pellets) and, most abundantly, as synthetic textile fibers shed during the laundering and wear of polyester and other synthetic fabrics, which constitute the single most commonly reported MP morphotype in freshwater and terrestrial matrices. These particles now occur across every environmental compartment that has been systematically sampled, including abyssal marine sediments, high-altitude glacial lakes, remote mountain springs, and the atmospheric boundary layer [4,5]. Beyond their physical persistence, MPs act as mobile reservoirs for co-contaminants such as polycyclic aromatic hydrocarbons (PAHs), polychlorinated biphenyls (PCBs), heavy metals, and endocrine-active additives, amplifying their toxicological significance across trophic levels [6,7].

Amphibians and reptiles occupy ecologically central but toxicologically distinctive positions in the terrestrial and freshwater biosphere. Amphibians respire and drink in part through a permeable, mucus-rich integument; most anurans and urodeles undergo biphasic life histories that straddle aquatic larval and terrestrial adult habitats; and both classes are ectothermic, tightly coupling physiological performance to ambient temperature and water quality [8,9]. These same traits render herpetofauna exceptionally responsive to emerging contaminants. Concurrently, approximately 41% of amphibian species and approximately 21% of assessed reptile species are classified as threatened on the IUCN Red List, with proportions exceeding 50% in turtles and crocodilians, placing herpetofauna among the most imperiled vertebrate radiations on Earth [10,11]. These pressures, habitat loss, climate change, invasive species, and the panzootic spread of chytridiomycosis now intersect with a rapidly growing MP burden, producing a convergent stressor landscape that current conservation frameworks were not designed to address. Recent integrative syntheses of herpetofaunal ecophysiology have shown that thermal, hydric, chemical, and pathogenic stressors interact synergistically rather than additively, frequently producing population-level impacts that exceed the sum of single-stressor effects [12]; MP contamination must therefore be evaluated within this multi-stressor framework rather than as an isolated agent.

Relative to fish and marine invertebrates, herpetofauna remain profoundly underrepresented in the ecotoxicological literature on plastic pollution. A systematic review dedicated to amphibian MP exposure identified only 41 studies through early 2022 [13], while plastic ingestion had been documented in all seven marine turtle species but in only five of 352 non-marine turtle species at the time of the most recent comprehensive assessment [14]. The orders Caudata and Gymnophiona, the majority of snake and lizard families, and all Crocodilia lineages remain almost entirely absent from ecotoxicological characterization. This deficit is particularly consequential because freshwater and soil matrices—primary habitats for most herpetofaunal species—function as net accumulation zones for MP pollution, receiving continuous inputs from surface runoff, atmospheric deposition, agricultural plasticulture, and inadequate waste management [15,16].

The ecological and evolutionary stakes of this exposure are substantial. Amphibians and reptiles together represent more than 18,000 extant species, encompass deep evolutionary lineages spanning more than 350 million years of independent territorialization, and contribute disproportionately to vertebrate phylogenetic diversity in tropical biodiversity hotspots. Many of the traits that define their evolutionary success—permeable integument optimized for cutaneous gas and water exchange in amphibians, cornified scales mediating water economy and chemical sensing in reptiles, biphasic life cycles, ectothermic metabolism, and behaviorally complex parental investment in some clades—also determine their differential exposure and physiological response to environmental contaminants. MP pollution therefore acts not as a single uniform stressor but as a phylogenetically structured selective pressure, with exposure intensity and toxicological consequence varying across lineages in ways that may have measurable effects on contemporary population dynamics, fitness variation, and microevolutionary trajectories. Framed in this way, MP contamination is not solely a conservation toxicology problem but an emerging axis of anthropogenic selection acting on populations whose evolutionary distinctiveness is itself a primary conservation value [12,17].

The present review addresses this fragmentation by integrating field and experimental evidence from five continents into a single, taxonomically balanced synthesis. Six objectives are pursued: (i) to characterize MP exposure pathways and bioaccumulation across herpetofaunal taxa and life stages; (ii) to evaluate the molecular and physiological mechanisms through which MPs impair development, behavior, and immunity in amphibians and reptiles; (iii) to quantify the role of trophic transfer and anthropogenic pressure gradients in shaping individual-level exposure risk; (iv) to consider how MP exposure intersects with herpetofaunal life-history and trait evolution, and how it might act as an emerging selective pressure on populations of conservation concern; (v) to identify hypothesis-driven research priorities that could move the field from description to mechanistic and evolutionary prediction; and (vi) to articulate the conservation and policy implications of current findings, including an evaluation of whether, and under what evidentiary conditions, MP burden might in the future be incorporated into IUCN threat assessments.

## 2. Scope and Approach

This article is presented as a structured narrative review. A narrative format was chosen because heterogeneity in analytical methods (visual identification, FTIR, Raman, pyrolysis-GC/MS), reporting units, size thresholds, and tissue targets across the herpetofaunal microplastics literature currently precludes statistically comparable effect-size synthesis, and because the broader goal identifying convergent ecological, mechanistic, and evolutionary patterns across phylogenetically diverse taxa is better served by integrative interpretation than by quantitative meta-analysis at this stage of the field.

Web of Science, Scopus, and PubMed were queried (publication window: January 2015—March 2026) using combined plastic-related terms (microplastic, nanoplastic, plastic debris, polymer particle, synthetic fiber) and herpetofaunal terms (amphibian, Anura, tadpole, urodele, caecilian, reptile, Squamata, lizard, snake, turtle, crocodile, herpetofauna); landmark earlier work was added through citation tracing. Sources were prioritized that combined peer review, primary empirical data on MP or NP exposure, ingestion, accumulation, or experimentally induced toxicity in any amphibian or reptilian life stage, and clear MP-identification methodology with spectroscopic confirmation (ATR-FTIR, Raman, or pyrolysis-GC/MS) preferred over visual classification alone. Synthesis was organized along two axes—taxonomic group and geographic region—and three asymmetries were noted as recurring constraints throughout: heterogeneous digestion and reporting protocols limit cross-study quantitative comparison and preclude derivation of harmonized effect concentrations; nanoplastic burdens in wild herpetofauna are almost certainly underestimated owing to analytical detection limits, particularly the vibrational-spectroscopy size cut-off near 10–20 µm; and laboratory exposures typically use pristine, uniform-size, single-polymer particles at concentrations exceeding environmentally realistic levels, complicating extrapolation to weathered field mixtures and limiting the usefulness of derived toxicity values for ecological risk characterization.

Because the present work is a narrative rather than a systematic review, it does not follow a PRISMA protocol; nevertheless, to improve methodological transparency, the screening workflow and selection criteria are stated explicitly. Records returned by the three databases were first de-duplicated and screened at the title and abstract level for relevance to MP or NP exposure in amphibians or reptiles. Full-text records were then assessed against the following inclusion criteria: (i) peer-reviewed primary research reporting MP or NP exposure, ingestion, tissue accumulation, or experimentally induced toxicity in at least one amphibian or reptilian life stage; (ii) identification of particles by morphology with, where possible, polymer confirmation by ATR-FTIR, Raman, or pyrolysis-GC/MS; and (iii) sufficient methodological detail (sample size, tissue analyzed, detection approach) to permit qualitative comparison. Records were excluded if they reported only environmental-matrix MP data without an organismal endpoint, lacked any particle-identification method, or were conference abstracts, opinion pieces, or non-peer-reviewed reports; secondary reviews were retained only for contextual framing and are identified as such in the text. Application of these criteria yielded a core evidence base of approximately 40 primary amphibian and reptilian field and experimental studies, supplemented by broader contextual literature on MP sources, environmental behaviour, and mechanistic toxi-cology in non-herpetofaunal models. A formal quantitative risk-of-bias scoring was not applied, consistent with the narrative format; instead, each primary study was appraised qualitatively for analytical rigor, prioritizing spectroscopically confirmed identifications and studies reporting procedural-blank and contamination-control (QA/QC) information, and findings derived from visual-only identification or from supra-environmental single-polymer exposures were flagged where they bear on interpretation.

## 3. Sources, Properties, and Environmental Behavior of Microplastics

### 3.1. Definitions and Classification

Microplastics are synthetic polymer particles with a maximum dimension below 5 mm [3]. They are categorized by origin into two broad classes. Primary MPs are manufactured at microscale for direct use as microbeads in personal care products, pre-production industrial pellets, and abrasive blasting media. Secondary MPs arise from the progressive physical, photochemical, and biological fragmentation of larger plastic items, including packaging, synthetic textiles, fishing gear, agricultural films, and vehicle tires [3]. Morphologically, MPs occur as fibers, fragments, films, pellets, and microbeads. Across environmental and biological matrices, fibrous particles are consistently the dominant morphotype, arising principally from the laundering of synthetic textiles, tire wear, and the abrasion of fishing nets and ropes [18]. This morphological dominance carries toxicological weight because fibers penetrate and compress gastrointestinal microvilli more effectively than spherical particles of comparable volume, producing greater epithelial damage and more pronounced intestinal dysbiosis [19].

Nanoplastics, conventionally defined as particles below 1 µm, although definitional consensus remains incomplete, may represent a distinct and, in several respects, potentially more hazardous fraction. It should be emphasized at the outset that the evidence base for nanoplastic-specific toxicity derives overwhelmingly from mammalian and in vitro models; direct experimental data for amphibians and reptiles remain largely absent, so the mechanisms summarized below are presented as plausible, hypothesis-generating extrapolations rather than as established findings for herpetofauna. Their markedly higher surface-area-to-volume ratio enhances sorption of chemical contaminants, and their small size permits translocation across biological barriers that exclude conventional MPs, including the gut epithelium, the blood–brain barrier, and, as demonstrated in mammalian models and inferred but not yet systematically verified in ectotherms, the placental or oviductal interface. In mammalian (mouse) hepatocytes, sub-micron polystyrene particles arriving through the circulation have been shown to induce nuclear and mitochondrial DNA damage, leading to cytosolic dsDNA leakage that activates the cGAS/STING DNA-sensing pathway and downstream NF-κB-driven inflammation, with chronic exposure progressing to liver fibrosis [20]; whether this mechanism operates analogously in amphibian or reptilian hepatocytes remains untested and constitutes a research priority. The near-universal exclusion of nanoplastics from environmental monitoring—an artefact of the detection limits of Fourier-transform infrared (FTIR) and Raman spectroscopy in complex biological matrices—represents a systematic blind spot in current risk assessment [21].

### 3.2. Polymer Types and the Trojan Horse Mechanism

The polymer families dominating environmental MP pools reflect those dominating global production: polyethylene (PE), polypropylene (PP), polystyrene (PS), polyvinyl chloride (PVC), polyethylene terephthalate (PET), and polyester fibers. It should be noted that most synthetic polyester textile fibers are themselves composed of PET; the terms “PET” and “polyester fiber” therefore overlap substantially and are not strictly independent polymer categories. Where the two are listed separately in this review, this reflects the reporting conventions of the original studies rather than a true chemical distinction. Density largely governs environmental fate. Low-density polymers such as PE and PP remain buoyant and concentrate at the air–water interface and in the surface pelagic zone, while higher-density polymers such as PVC and PET tend to settle into sediments. These partitioning patterns translate directly into differential exposure across herpetofaunal taxa and life stages: filter-feeding tadpoles and surface-skimming juvenile sea turtles encounter buoyant polymers, whereas benthic amphibian larvae, freshwater turtles, and fossorial squamates contact a heavier, sediment-bound polymer fraction [4].

**Figure 1:**
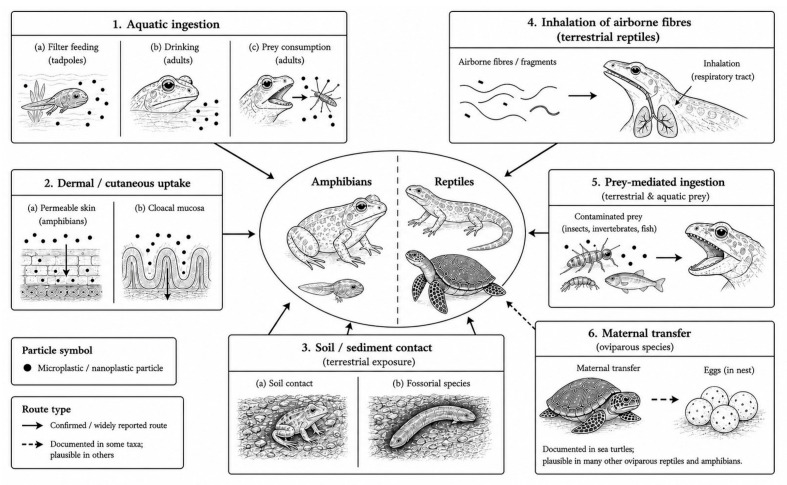
Conceptual diagram of microplastic exposure routes in amphibians and reptiles. The amphibian integumentary pathway is shown separately from pathways relevant to reptiles. The six numbered panels denote distinct exposure routes: (1) aquatic ingestion (filter feeding in tadpoles, drinking and prey consumption in adults); (2) dermal/cutaneous uptake across the permeable skin and cloacal mucosa of amphibians; (3) soil and sediment contact during terrestrial and fossorial activity; (4) inhalation of airborne fibres in terrestrial reptiles; (5) prey-mediated (trophic) ingestion of contaminated invertebrate and vertebrate prey; and (6) maternal transfer to eggs in oviparous species (documented in sea turtles and considered plausible in many other oviparous reptiles and amphibians). Arrow style indicates the strength of evidence for each route rather than the exposure medium: solid arrows denote confirmed or widely reported routes, and dashed arrows denote routes documented in some taxa but still considered plausible or less firmly established in others (e.g., maternal transfer). Filled circles represent microplastic and nanoplastic particles generically and do not encode specific polymer identities.

The toxicological potential of MPs is not confined to the polymer matrix. Two chemical dimensions amplify their hazard profile. First, manufacturing additives, plasticizers such as phthalates, UV stabilizers, brominated and organophosphorus flame retardants, and metal-based colorants are embedded in the polymer and leach over time under physiological conditions. Second, MPs sorb hydrophobic organic pollutants (PAHs, PCBs, organochlorine pesticides) and trace metals from the surrounding water or soil with high affinity and can transport them across long distances [6,7]. Environmental aging of MP particles through ultraviolet radiation, mechanical abrasion, hydrolytic weathering, and biofilm colonization progressively modifies their surface chemistry, increasing both effective surface area and the density of oxygen-containing functional groups, with consequent elevation of sorption capacity for hydrophobic organic contaminants and divalent metal cations relative to pristine virgin particles [6,7]. This temporal evolution of particle reactivity has a clear practical consequence. Field-aged MP burdens dominate the matrices that free-living herpetofauna actually encounter. These weathered particles therefore deliver exposure profiles toxicologically distinct from those of the pristine particles used in most laboratory tests. The resulting mismatch limits the predictive validity of laboratory-derived effect concentrations for field-realistic risk. On ingestion, the acidic and enzymatic environment of the gastrointestinal tract promotes desorption of these chemical passengers, increasing their local bioavailability by orders of magnitude relative to dilute ambient concentrations. This “Trojan horse” mechanism means that MPs deliver a concentrated chemical payload to organisms that would rarely encounter those compounds at biologically meaningful concentrations in the external environment—a reality that standard ecotoxicity testing on pristine, uncontaminated polymer particles systematically fails to capture (Figure 1).

## 4. Microplastic Exposure and Bioaccumulation in Amphibians

### 4.1. The Larval Stage as the Primary Exposure Target

Tadpoles represent the amphibian life stage most intensively exposed to MP contamination in freshwater ecosystems. Shallow, small lentic waterbodies—the dominant breeding habitat for most anuran species—act as hydrological sinks, receiving disproportionately high inputs from surface runoff, atmospheric deposition, and stormwater discharge. Within intensively urbanized catchments, MP concentrations in these small waterbodies can exceed surface oceanic levels by factors of 4–23, though this ratio is strongly context-dependent and should not be extrapolated to rural or remote freshwater systems [22]. The combination of elevated ambient concentrations and the indiscriminate suspension-feeding behavior of larval anurans, which captures particles across a broad size range, produces exposure conditions that are structurally distinct from those experienced by most other freshwater vertebrates.

Reported MP detection prevalence in larval amphibians spans a remarkably wide range. At the lower end, a conservative size threshold applied to a ten-species Central European survey detected MPs in 26% of 201 tadpoles [23]. At the upper end, larvae of *Pelophylax* spp. from Anatolian catchments under high anthropogenic pressure carried particles in 73–80% of examined individuals [24]. This variance is driven principally by genuine ecological heterogeneity rather than by methodological artefact alone: a positive, quantitatively resolvable relationship between catchment-level anthropogenic pressure indices and individual-level MP burden has been documented using comparable protocols across multiple Anatolian and European systems [25]. Across studies, fibrous particles dominate the detected MP pool, accounting for 57–91% of particles identified in anuran larvae, with PET, polyester, and PCT (poly [1,4-cyclohexylenedimethylene terephthalate], a polyester-class terephthalate polymer) repeatedly emerging as the most prevalent polymers [25,26]. This fiber predominance is toxicologically consequential given the superior capacity of fibers to penetrate intestinal microvilli and induce epithelial compression relative to spherical particles of comparable volume.

Tadpoles acquire MPs through at least three coexisting pathways. Direct ingestion captures water-column particles via filter-feeding and buccal suction. Indirect ingestion occurs through consumption of periphyton biofilms in which MPs are embedded, a pathway of increasingly appreciated quantitative importance for freshwater grazers [27,28]. A third route, through ingestion of MP-laden benthic detritus, becomes important in later developmental stages as tadpoles shift toward benthic foraging. The relative contribution of each pathway varies with species, developmental stage, and the physical characteristics of the waterbody. Critically, MPs entering the larval gut follow one of two fates: egestion before or during metamorphic gut remodeling, or retention in somatic tissues with the potential to transfer to the post-metamorphic adult and, through predation, to higher trophic levels.

### 4.2. Adult Amphibians: Multiple Simultaneous Exposure Routes

Post-metamorphic amphibians have received substantially less attention than larvae, yet the evidence that exists reveals simultaneous exposure across multiple routes. A pioneering multi-route study on two Amazonian anuran species (*Physalaemus ephippifer* and *Boana multifasciata*) documented MP contamination concurrently in the digestive tract, the respiratory tract, and the integument [29]. This pattern underscores a structural feature of the amphibian body-plan: a highly permeable, mucus-rich skin that facilitates gas exchange and cutaneous water absorption simultaneously creates an MP entry pathway entirely absent in amniotic vertebrates.

Ecological specialization further modulates exposure. In arboreal hylids such as *Boana multifasciata*, leaf surfaces function as a secondary exposure substrate, with MP particles adhering to cuticular waxes and subsequently ingested during licking or prey capture behavior, a foliar exposure pathway first proposed for amphibians on the basis of these observations [29,30]. In strictly terrestrial species with limited aquatic connectivity, soil-bound MPs, particularly fibers originating from nearby agricultural or urban land use, become the principal exposure reservoir, accessed through inadvertent soil ingestion during prey capture [16].

An unexpected intra-population pattern has emerged from post-metamorphic surveys. In *Amietia delalandii* from South Africa, larger individuals carried fewer and shorter MP fibers than smaller conspecifics [31]. This negative body size–MP burden relationship may reflect selective prey-size preferences, enhanced physiological clearance in larger individuals, or differential particle retention kinetics across body-size classes. Whatever its mechanistic basis, it has direct implications for how exposure risk is distributed across age classes within wild populations and argues against the implicit assumption, common in the toxicological literature, that larger individuals necessarily bear larger contaminant loads. More generally, the influence of host age, sex, and body size on MP uptake remains inconsistently characterized in herpetofauna and deserves explicit attention in future sampling designs. The available evidence is mixed: the negative body-size relationship reported above contrasts with the absence of any significant age- or sex-related difference in MP burden documented in adult Siberian wood frogs [32], while broader freshwater data (e.g., in co-occurring fish) suggest that ontogenetic shifts in diet and feeding rate, rather than body size per se, drive much of the variation. Sex-specific differences, where examined, are generally weak and may be confounded by body-size dimorphism and by reproductive-state effects on feeding. These patterns indicate that age, sex, and body size should be recorded and analyzed as covariates rather than assumed to scale monotonically with exposure.

Beyond the anurans, the Urodela and the Gymnophiona remain comparatively neglected. Studies on *Ommatotriton* newts (*O. nesterovi*, *O. vittatus*, *O. ophryticus*) detected MPs in 29–43% of 91 adult salamanders, providing among the first detailed characterizations of MP ingestion in post-metamorphic urodeles [33]. Endemic Turkish *Neurergus* species (*N. barani*, *N. strauchii*), despite occupying remote montane spring habitats with minimal direct anthropogenic contact, exhibited detectable PET contamination—direct field evidence that atmospheric deposition and upstream transport are capable of delivering MPs to geographically isolated populations [34]. The Gymnophiona, comprising more than 200 described species, remain wholly absent from the MP exposure literature—a striking omission given their fossorial habits and consequently high soil-contact exposure.

### 4.3. Geographic Patterns of Exposure

Europe and the Mediterranean basin remain the best-characterized regions for amphibian MP exposure. A multi-species larval survey across 10 anuran species in Poland detected MPs in 26% of 201 tadpoles, with 71 distinct particle types identified and a mean of 3.2 particles per individual in Bufonidae larvae [23], and a broader European comparative analysis documented fiber predominance in *Rana arvalis* (61%), *Rana temporaria* (57%), and the *Pelophylax esculentus* complex (62%) alongside a positive correlation between anthropogenic pressure and MP load [25]. In the Mediterranean basin, a spatiotemporal analysis of *Pelophylax* species (*P. ridibundus*, *P. bedriagae*, *P. caralitanus*) across 11 Anatolian localities demonstrated a steep urban-to-rural gradient; MPs were absent from roughly 64% of remote-site samples yet substantially elevated in metropolitan Istanbul catchments [24], and inter-specific sensitivity differences were documented in parallel exposure experiments on *Rana latastei* and *Bufotes balearicus*, with *R. latastei* more vulnerable than *B. balearicus* under equivalent treatment [35].

Research across the Asia-Pacific, particularly in China and Thailand, has documented high exposure levels in both freshwater and terrestrial amphibian habitats. A study of tadpoles from small waterbodies in the Yangtze River Delta recorded freshwater MP concentrations substantially exceeding surface oceanic levels, framing rapidly urbanizing Asian catchments as critical MP accumulation zones [22]. A comprehensive multi-taxon assessment in western Thailand detected MPs in 91.67% of examined terrestrial vertebrate species, including 32.25% of salamander individuals—among the highest prevalence rates so far reported for this order, with contamination significantly higher outside protected-area boundaries [36].

South American work has extended the known geography of amphibian MP exposure into tropical rainforest ecosystems. The documentation of MP contamination in adult *Physalaemus ephippifer* and *Boana multifasciata* from the Amazon basin establishes that MP pollution is no longer confined to degraded or peri-urban environments but has now penetrated one of the last large wilderness regions on Earth [29,37]. In North America, experiments on *Rana sylvatica* have addressed MP–parasite interactions, with polyester microfiber ingestion demonstrated to modify trematode transmission dynamics, plausibly through altered mucus secretion and feeding behavior [38]. Trophic-transfer experiments on *Physalaemus cuvieri* larvae in Brazil documented measurable MP movement through a tadpole–fish–mammal chain, with behavioral impairments, reduced locomotion, and elevated anxiety indices in the mammalian recipients [39].

African amphibian MP research is scarce but ecologically informative; beyond the body-size–MP burden relationship documented in *Amietia delalandii* [31], the continent’s extraordinary amphibian diversity—concentrated in the Eastern Afromontane, the Guinean Forests, and the Cape Floristic Region—remains essentially uncharacterized, despite several African nations ranking among the highest per-capita contributors to riverine plastic discharge [40].

The most striking recent finding is the detection of MPs in amphibian populations inhabiting geographically remote, high-altitude habitats previously considered insulated from anthropogenic pollution. The endemic Turkish *Neurergus* species, restricted to clear-water montane springs, exhibited MP contamination dominated by PET, with discarded plastic water bottles within the catchment identified as the most plausible proximal source [34]. Marsh frogs (*Pelophylax ridibundus*) from remote Anatolian lakes carried lower but demonstrably non-zero burdens, attributable to atmospheric deposition and localized tourism inputs [41]. A comparative study across *Hyla orientalis* and *Hyla savignyi* populations documented substantially higher MP prevalence in *H. savignyi* (56.5%) than in *H. orientalis* (11.8%) from equivalent geographic regions, suggesting that species-specific niche use, microhabitat selection, and feeding ecology can modulate exposure even under shared regional contamination [42]. Beyond the Mediterranean and European literature, the first evidence of MP ingestion in a wild amphibian from the Russian Federation comes from the Siberian wood frog (*Rana amurensis*) in the Western Baikal region, where particles—predominantly microfibers (84.6%)—were detected in the gastrointestinal tracts of 83% of adults at a mean of 3.5 ± 3.6 particles per individual; notably, no significant relationship was found between MP burden and the age or sex of the frogs, and no particles were recovered from larvae sampled from low-productivity floodplain breeding pools [32]. This Siberian record extends the documented range of herpetofaunal MP exposure across northern Eurasia and reinforces that remote, sparsely populated catchments are not exempt from contamination. Taken together, the available studies suggest that, in the systems examined to date, geographic isolation from industrial activity does not necessarily guarantee refuge from MP contamination; given the still-limited number of remote-habitat studies, this should be treated as a tentative pattern rather than a firmly established global conclusion (Table 1).

## 5. Toxicological Effects of Microplastics in Amphibians

Interactions between MPs and amphibian physiology unfold simultaneously across molecular, cellular, organ, behavioral, and population levels. The breadth and severity of these effects reflect a combination of traits—permeable integument, biphasic life history, endocrine sensitivity during metamorphosis, and ectothermic metabolic physiology—that is not fully recapitulated in any other vertebrate class and that renders amphibians exceptionally useful sentinels of emerging contaminant pressure [9,45].

### 5.1. Growth and Development

**Figure 2:**
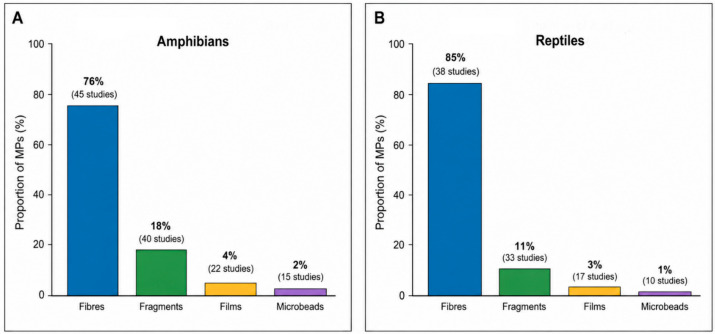
Compositional predominance of fibrous microplastics in amphibians and reptiles, based on pooled study averages. (**A**) Mean morphological composition of microplastics reported in amphibian field studies; (**B**) mean morphological composition reported in reptile field studies. In both groups, fibres are the dominant morphotype. Values are unweighted arithmetic means of the percentage composition reported in the field studies compiled in Table 1 and Table 3 that provided a morphological breakdown (*n* = 11 amphibian and 8 reptile studies); because the underlying studies differ in digestion protocol, size threshold, and identification method, the figure illustrates the qualitative predominance of fibres rather than a quantitatively weighted estimate.

During embryogenesis, MPs exert concentration-dependent embryotoxic and teratogenic effects. In *Xenopus laevis*, polyethylene microplastics (PE-MPs) elicit dose-dependent increases in both embryonic mortality and malformation frequency, including cardiac edema, axial deformity, and notochord abnormalities, each of which compromises larval viability and post-hatching survival [46]. Mechanistic dissection implicates both reactive oxygen species (ROS) overproduction, perturbing redox-sensitive developmental signaling, and direct physical interference with embryonic cell division through entrapment of particles within developing tissues. After hatching, active suspension feeding exposes larvae to ingestion rates that scale proportionally with both particle abundance and feeding intensity (Figure 2).

In *Rana zhenhaiensis*, exposure to PE and polystyrene microplastics (PS-MPs) produced significant, concentration-dependent reductions in body mass, body length, and hindlimb length, a composite effect attributable to mechanical obstruction of the gastrointestinal tract, epithelial injury reducing nutrient absorption, and interference with growth hormone signaling [47]. Consistent findings emerged from *Alytes obstetricans*, where increasing MP concentrations reduced feeding rate, impaired growth trajectories, and elevated mortality at higher doses [43]. Particle morphology strongly modulates severity: irregular polyester fibers produce substantially greater intestinal injury than spherical particles of equivalent volume, penetrating microvilli, compressing epithelial tissue, and inducing dysbiosis [19]. Tire wear particles (TWPs), which carry a distinct chemical cargo including 6PPD-quinone and zinc compounds, caused severe morphological abnormalities and elevated mortality in *Silurana tropicalis* at environmentally realistic concentrations, reinforcing the lesson that particle source and chemistry are as toxicologically consequential as size and shape [9].

The developmental response is not uniformly suppressive. In *Rana sylvatica*, certain MP concentrations have been associated with paradoxical increases in larval mass, attributable to lipid-metabolism disruption by plastic-associated chemicals producing an “obesogenic” physiological state rather than genuine improvement in body condition [44]. In *Xenopus laevis*, specific exposure scenarios accelerate metamorphic timing and elevate metabolic rate, leaving juveniles with altered morphology, suggesting that MP exposure shifts developmental trajectories in multiple directions depending on polymer chemistry, concentration, and stress history [48]. This bidirectionality complicates single-endpoint risk assessment and argues for multi-parameter developmental toxicity testing protocols.

At metamorphic climax, MPs interfere with endocrine regulation, particularly the hypothalamic–pituitary–thyroid (HPT) axis, through mechanisms detailed in Section 5.5. The resulting metamorphic disruption is bidirectional: delay in *Rana sylvatica* [44] and acceleration in *Xenopus laevis* [48], each carrying distinct ecological fitness costs. “Legacy effects”, persistent physiological impairments that carry through the metamorphic transition, reduce post-metamorphic survival and fitness even when juveniles are subsequently housed in uncontaminated environments [9,44], redefining MP exposure as a driver of lifetime fitness reduction rather than a discrete larval stressor.

### 5.2. Behavioral and Physiological Effects

Behavioral changes are among the earliest and most ecologically consequential manifestations of MP toxicity in amphibians because they directly degrade survival-critical functions: predator avoidance, foraging, and social signaling. In *Physalaemus cuvieri* tadpoles, even short-term exposure reduces locomotor activity, suppresses exploratory behavior, and weakens anti-predator escape responses, impairments that would translate directly into elevated predation mortality in natural populations [39]. In *Xenopus laevis*, fiber exposure reduces swimming distance and velocity, degrading both foraging capacity and predator evasion [19]. In *Rana latastei*, reduced anti-predator responsiveness co-occurs with growth suppression, implying shared neuromuscular mechanisms linking behavioral and physiological outcomes [49].

At the physiological level, MP exposure triggers a coordinated stress response. Elevated corticosterone concentrations—the principal glucocorticoid mediating stress responses in amphibians—have been documented following MP exposure, accompanied by upregulation of antioxidant enzymes, including superoxide dismutase (SOD) and catalase [50]. While initially adaptive, chronic activation of these systems depletes antioxidant reserves and diverts energy from growth and reproduction toward cellular maintenance. Inhibition of acetylcholinesterase (AChE) and butyrylcholinesterase (BChE) implicates MPs as direct disruptors of cholinergic neurotransmission, providing a mechanistic bridge to the locomotor impairments observed behaviorally [50].

The gastrointestinal tract is a primary target of MP-induced pathology, particularly for fibrous particles. In *Xenopus laevis* larvae, microfiber accumulation along the intestinal lining triggers compensatory increases in gut length and mass, a response to reduced nutrient extraction efficiency [19]. However, this adaptive remodeling has limits: prolonged polyester fiber exposure causes architectural deformation of the gut, epithelial compression, and, in severe cases, physical penetration of fibers through the gut wall or complete luminal obstruction [51]. These gastrointestinal pathologies reduce assimilation efficiency, compromise the mucosal immune barrier, and, in conjunction with microbiome disruption, create conditions that favor opportunistic pathogen proliferation.

### 5.3. Cellular and Molecular Toxicity

At the tissue level, MP exposure produces histopathological change in multiple organ systems, with liver and gut emerging as primary targets. In *Rana zhenhaiensis*, PE and PS exposure altered hepatic parenchymal architecture, including expansion of intercellular spaces and increased melanomacrophage aggregate abundance, hallmarks of impaired detoxification and chronic hepatic stress [47]. Under extended exposure, these changes can progress to fibrotic remodeling with loss of functional hepatocyte architecture, a trajectory mechanistically characterized in mammalian models [20] and inferred but not yet directly demonstrated in amphibians. At the intestinal level, fibrous particles disrupt enterocyte integrity in *Xenopus laevis*, producing cell detachment and epithelial rupture and degrading both absorptive and immunological function [19].

At the cellular level, MP-induced cytotoxicity combines direct physical damage with ROS-mediated oxidative injury. In *Physalaemus cuvieri* tadpoles, environmentally relevant PE concentrations (60 mg L^−1^) induced erythrocyte cytotoxicity, micronuclear genotoxicity, and external morphological abnormality [52]. Systemic distribution of MPs documented across gill, gut, liver, tail muscle, and peripheral blood confirms translocation across biological barriers as a consistent feature of MP exposure in amphibians and raises the probability of multi-organ dysfunction.

Molecular mechanisms converge on oxidative stress as a central upstream pathway. Excessive ROS generation drives DNA strand breaks in both nuclear and mitochondrial compartments, activates lipid peroxidation, and precipitates mitochondrial dysfunction [53]. The intracellular signaling cascade described next has been characterized only in mammalian and in vitro systems and has not yet been demonstrated in amphibians or reptiles; it is therefore presented as an inferred, hypothesis-generating mechanism rather than an established herpetofaunal pathway. In mammalian hepatocyte models, sub-micron particles entering from circulation have been shown to activate the cGAS/STING cytosolic DNA-sensing pathway and downstream NF-κB translocation to the nucleus, upregulating pro-inflammatory cytokines, including TNF-α, IL-1β, and IL-6 [20]. Whether this cascade, implicated in driving hepatic stellate cell activation and fibrogenesis under chronic exposure in mice, translates mechanistically to amphibian hepatocytes remains to be tested experimentally. Transcriptomic analyses further reveal dysregulation in gene networks governing lipid metabolism, immune function, and apoptotic regulation. In *Xenopus laevis*, MP exposure disrupts key immune-related genes, including IL-34 and MHC-II, indicating that homeostatic disruption extends into the adaptive immune compartment [54,55].

### 5.4. Disease Susceptibility and Immune Disruption

MP pollution has emerged as a quantitatively important modulator of amphibian disease dynamics, acting principally through disruption of multiple tiers of host immune defense. The mechanistic cascade operates through at least three convergent pathways. First, MP ingestion disturbs the gut microbiome, reducing populations of beneficial commensals that provide colonization resistance, prime mucosal immune responses, and stimulate antimicrobial peptide (AMP) production. Second, MP particles internalized by macrophages and other phagocytes occupy cellular processing machinery, limiting the phagocytic capacity available for pathogen clearance. Third, chemical additives and co-contaminants released from ingested MPs suppress lymphocyte proliferation and innate immune signaling, producing a generalized immunodysregulation [56]. This pattern is consistent with broader evidence that environmental stressors activating the hypothalamus–pituitary–interrenal (HPI) axis suppress both innate and lymphocyte-mediated immune responses in herpetofauna, increasing susceptibility to chytrid fungi and ranaviruses [12], placing MP-mediated immunosuppression within an established physiological framework for stressor–pathogen interactions in amphibians.

The clearest consequences appear in the context of viral pathogens. In *Xenopus laevis* tadpoles, PET exposure reduces antiviral immunity and significantly elevates susceptibility to ranavirus FV3, a major driver of amphibian mass mortality worldwide [56]. The mechanism involves impaired macrophage function and disrupted cytokine signaling necessary for effective antiviral response. This finding carries substantial conservation weight given that ranavirus outbreaks have precipitated population collapses across multiple species, and that MP contamination now co-occurs with ranavirus presence across the geographic range of susceptible taxa.

For fungal pathogens, the interaction between MP exposure and *Batrachochytrium dendrobatidis* (Bd)—the causative agent of chytridiomycosis and the most destructive pathogen ever documented in vertebrates—warrants particular emphasis. Preliminary experimental evidence indicates a dose-dependent increase in Bd infection load with increasing MP concentration, consistent with a potential synergistic interaction between pollutant stress and pathogen virulence [44,57], although confirmation across additional species and exposure regimes is required before generalization. The most plausible mechanism combines MP-induced dysbiosis of the cutaneous microbiome, which in healthy amphibians includes Bd-antagonistic bacterial taxa such as *Janthinobacterium lividum*, with physical disruption of the skin mucus layer that normally impedes Bd zoospore attachment. If confirmed, this convergent vulnerability could mean that species already experiencing chytridiomycosis-driven decline face additional disease pressure in MP-contaminated environments. It must be stressed, however, that this remains a mechanistically plausible hypothesis: the supporting experimental evidence is still preliminary and largely limited to single-species dose–response observations, and the magnitude of any interaction has not yet been quantified.

### 5.5. Endocrine Disruption and the HPT Axis

MP-mediated endocrine disruption in amphibians operates through two mechanistically distinct but functionally convergent routes. The first involves chemical additives, particularly bisphenol A (BPA) and phthalate esters, acting as xenoestrogens and antiandrogens that bind estrogen, androgen, and other nuclear hormone receptors with sufficient affinity to reconfigure gene expression programs governing gonadal differentiation, gamete production, and reproductive behavior, with documented consequences including gonadal intersex, skewed sex ratios, and compromised reproductive fitness in multiple anuran species. The second pathway operates in a neuroendocrine manner on the hypothalamic–pituitary–thyroid (HPT) axis: plastic-associated contaminants such as polybrominated diphenyl ethers (PBDEs), PCBs, and specific phthalates competitively inhibit thyroid hormone synthesis, displace T4 from transthyretin, and block thyroid hormone receptor binding, with histological correlates documented in MP-exposed *Xenopus laevis* as follicular hyperplasia and disorganized colloid storage [9,54].

HPT axis disruption is especially severe in amphibians, for whom thyroid hormones are not merely metabolic regulators but the primary endocrine drivers of metamorphosis. Suppression of T3/T4 signaling delays or arrests metamorphic climax, extending larval-period duration and associated predation and desiccation risk, while certain endocrine-active compounds conversely accelerate metamorphosis, producing juveniles with insufficient somatic reserves for terrestrial establishment [44,48]. The resulting shifts in metamorphic timing decouple seasonal emergence from prey availability and may interact additively with climate-driven phenological shifts; “endocrine legacy effects” can persist post-metamorphically as altered stress-axis reactivity and reduced immunocompetence even after exposure ceases (Table 2).

## 6. Microplastic Exposure and Bioaccumulation in Reptiles

Reptiles encounter MPs across aquatic, freshwater, and terrestrial habitats through three primary routes: direct ingestion of contaminated water and soil, secondary ingestion through MP-laden prey, and, for species occupying specific microhabitats, inhalation of airborne fibers [58,59]. The ecophysiology of reptiles is sufficiently distinct from that of amphibians to anticipate differing bioaccumulation kinetics. Longer gut retention times relative to endothermic vertebrates, dietary breadth spanning strict carnivory to herbivory, and ectothermic metabolic rates that may slow particle clearance combine to produce taxon-specific accumulation dynamics. Once internalized, MPs exert physical effects such as gastrointestinal obstruction, mucosal abrasion, and caloric dilution and chemical effects through additive leaching and co-contaminant desorption in the digestive environment [60]. At the molecular level, MPs in reptiles induce oxidative stress, cytotoxicity, and elevated TNF-α, IL-1β, and IL-6 expression, indicating systemic inflammatory activation comparable to that documented in amphibians [21,58].

### 6.1. Sea Turtles: The Best-Studied Reptilian Group

Sea turtles have attracted the bulk of reptilian MP research effort, with plastic ingestion documented in all seven extant species across multiple life stages and ocean basins [61,62]. The pelagic juvenile “lost years” phase, during which juveniles drift passively with surface currents and consume floating organic material, coincides with the highest densities of buoyant plastic debris in oceanic surface convergence zones; in post-hatchling sea turtles from Florida coastal waters, plastic particle ingestion has been documented in up to 92.86% of individuals [63], and juvenile *Chelonia mydas* from Galápagos and Machalilla National Park (Ecuador) exhibited MP prevalence approaching 98%, with fibrous PE/PP particles predominating [64]. Beyond simple gastrointestinal accumulation, high-resolution organ analyses in Mediterranean *Caretta caretta* have detected MP particles across virtually every examined visceral organ—heart, liver, kidney, and reproductive tissues—with the highest mass concentrations in cardiac and reproductive compartments [65]; the detection of MPs in reproductive tissue is especially consequential because it represents the anatomical prerequisite for maternal transfer to developing embryos. Even modest ingested plastic quantities—particularly of soft, compressible materials that resist gastric passage and accumulate into obstructive masses—can be lethal [1].

### 6.2. Maternal Transfer and the Nesting Beach as an Exposure Arena

The detection of MPs within sea turtle eggs constitutes one of the most ecologically alarming recent findings in herpetofaunal MP research because it demonstrates that plastic contamination can compromise organismal fitness before hatching. In a systematic survey of non-viable *Caretta caretta* eggs from northwest Florida nesting beaches, MPs were present at every sampled site, with 494–510 particles identified across 350 eggs from 70 nests, a mean of 1.46 particles per egg [66]. Fibers dominated at 89.8% of total particles, and polypropylene was the most prevalent polymer at 34.6%. The presence of MPs within eggs implies maternal transfer through either circulatory deposition into developing follicles or post-ovulation translocation across the oviductal interface, a pathway substantiated by the documented occurrence of MPs in the reproductive organs of adult females [65] and by parallel evidence for maternal transfer of persistent organic pollutants in sea turtles [67].

Nesting beach sediments now constitute an external MP exposure reservoir of global significance. Comprehensive global assessments confirm MP presence on marine turtle nesting beaches worldwide, with concentrations reaching thousands of particles per kilogram of dry sand across multiple sediment depth layers [68]. The physical presence of MPs alters the thermal, hydric, and gaseous microenvironment of nests by changing sand thermal conductivity, modulating moisture retention and desiccation rates, and potentially restricting oxygen diffusion to incubating eggs [69]. These physical modifications carry direct developmental consequences. Because sea turtles exhibit temperature-dependent sex determination, alterations in nest thermal profiles shift hatchling sex ratios—an outcome already of acute conservation concern under climate warming projections [61]. MP-induced changes that elevate nest temperatures would further amplify female-biased sex ratios, threatening long-term population viability. Concurrently, chemical contaminants sorbed to MP particles, including PCBs and dioxins, can diffuse into the egg environment during incubation, producing embryonic chemical toxicant exposure independent of maternal transfer.

### 6.3. Freshwater Turtles: High Endangerment, Limited Research Attention

Freshwater turtles are, by species count, the most threatened chelonian group globally, with endangerment rates exceeding those of marine turtles. Yet their representation in MP research is inversely proportional to their conservation urgency. A systematic review identified confirmed plastic ingestion in only five of 352 non-marine turtle species [14], a striking research gap for a group in which more than half of species face extinction risk. This neglect is particularly concerning given that freshwater ecosystems function as primary MP accumulation zones, receiving continuous inputs from terrestrial and urban sources through riverine transport, stormwater, and atmospheric deposition [15].

Where evidence does exist, it confirms genuine exposure. In *Trachemys scripta elegans*, 7.7% of surveyed individuals had ingested plastic, including PE, PP, PET, and PS fragments, though identification challenges in the absence of spectroscopic confirmation introduce uncertainty into prevalence estimates [14]. Reported biological consequences include feeding inhibition, reduced growth and survival, and systemic tissue accumulation. Broader ecotoxicological assessments suggest that MPs may induce oxidative stress, activate innate immune responses, and produce tissue-level pathology in freshwater turtles [21,59], although direct mechanistic evidence remains limited for this group. Given the acute conservation status of freshwater chelonians and the documented MP loading of freshwater habitats, the ecological risk posed to this group is almost certainly underestimated by current data.

### 6.4. Terrestrial and Freshwater Reptiles: A Global Perspective

MP pollution is increasingly recognized as a relevant stressor for reptile biodiversity across terrestrial and freshwater habitats, extending well beyond the marine systems that have historically dominated research attention. The dominant ingested forms across terrestrial and freshwater reptilian taxa are fibrous particles composed predominantly of PET, PP, and PE, a pattern consistent with amphibian data and reflecting the environmental ubiquity of synthetic textile-derived fibers as the most abundant MP morphotype globally [58,70] (Table 3).

The Mediterranean basin functions as a global hotspot for terrestrial reptile MP exposure, with contamination levels strongly correlated with anthropogenic land use intensity. In Türkiye, MPs have been documented in multiple endemic and non-endemic reptile species. The endemic Cappadocian rock lizard (*Apathya cappadocica*) exhibited an MP prevalence of 19.35%, with polyvinyl alcohol (PVA) fibers predominating [71], while the Turkish worm lizard (*Blanus strauchi*) showed an ingestion prevalence of 24.57%, indicating that contamination penetrates even the soil horizons occupied by fossorial species [58].

Long-term temporal analyses using archived museum collections provide a historical perspective on contamination trajectories. Analysis of *Ophisops elegans* specimens collected between 1986 and 2013 detected MPs in 8.3% of 300 individuals, with habitat type rather than collection year emerging as the primary determinant of contamination, suggesting that land-use context exerts a stronger influence than pollution timeline on individual exposure [72]. In contrast, dice snakes (*Natrix tessellata*) and grass snakes (*Natrix natrix*) collected between 1986 and 2019 showed substantially higher contamination rates of 51.9% and 57.1%, respectively, with PET fibers constituting 87.9–94.7% of identified particles [73]. The elevated contamination in semi-aquatic colubrids relative to fully terrestrial lizards reflects their greater dietary dependence on aquatic prey and consequent exposure to MP-contaminated freshwater food chains.

Across the Asia-Pacific, high MP exposure has been documented in a taxonomically diverse array of reptilian taxa. Sea snakes in the United Arab Emirates exhibited MP presence in 95.3% of examined individuals, with nylon fibers—a polymer indicative of fisheries and aquaculture waste—dominating and implicating wastewater discharge from fishing operations as the primary regional source [21]. In Thailand, a comprehensive multi-species terrestrial assessment detected MPs in 44.12% of snakes, with individuals outside protected areas showing a numerically higher prevalence than those within protected zones, although the difference did not reach statistical significance, suggesting that even formally designated landscapes provide limited protection against MP infiltration [36]. In mangrove-associated habitats, the dog-faced water snake (*Cerberus rynchops*) carried an average of 5.96 MPs per individual, with polyester and PET fibers dominating, consistent with mangrove ecosystems functioning as effective traps for riverine MP transport.

In North American freshwater systems, *Trachemys scripta elegans*, the red-eared slider, widely invasive globally, has been confirmed as an MP accumulator across multiple study populations [14], and sea turtle nesting beaches along the Atlantic and Gulf coasts show widespread MP presence with PE as a dominant polymer, reflecting regional packaging waste inputs [68].

Latin American and Caribbean data remain limited but increasingly document pervasive contamination across ecologically diverse reptilian taxa: first evidence of MPs in invasive geckos (*Hemidactylus mabouia* and *H. angulatus*) in Cuba established both direct ingestion and trophic transfer as operational exposure routes for insular Caribbean herpetofauna [74]. Despite the region accounting for approximately 8% of global plastic consumption [75], only ~5% of global MP studies originate from Latin America, making this combination of high reptilian biodiversity and intensive plastic pollution pressure a high-priority research target [76]. A comparable geographic gap exists for northern Eurasia. Despite the recent documentation of MP ingestion in a Siberian amphibian [32] and in remote-river Siberian freshwater fish [77], our structured search did not identify any peer-reviewed study quantifying MP ingestion in wild Russian or Siberian reptile (snake or lizard) populations that met the inclusion criteria. We therefore note the absence of such data as a specific regional knowledge gap rather than inferring a contamination status for these populations; the freshwater evidence from the same region [77] indicates that the food webs supporting semi-aquatic reptiles there are already MP-contaminated, which makes targeted reptile surveys in Russia and Siberia a clear priority.

**Table 3:** Summary of microplastic contamination studies in reptiles, organized by habitat type, geographic region, and taxonomic group. As for Table 1, identification methods and lower size thresholds differ among the compiled reptile studies; contamination values are individual-level detection prevalence where reported, and cross-row comparison should be treated as qualitative.

Species/Group	Habitat	*n*	Contamination (%)	Dominant Polymer(s)	Reference
*Caretta caretta*	Marine	121	100	Elastomers, fibers	[62]
*Chelonia mydas*	Marine	102	100	Fibers (80.7%)	[62]
*Chelonia mydas* (juv.)	Marine	n/a	≈98	PE, PP, fibers	[64]
*Natrix natrix*	Freshwater	14	57.1	PET fibers (94.7%)	[73]
*Natrix tessellata*	Freshwater	27	51.9	PET fibers (87.9%)	[73]
*Ophisops elegans*	Terrestrial	300	8.3	Fibers	[72]
*Apathya cappadocica*	Terrestrial	n/a	19.35	PVA fibers	[71]
*Blanus strauchi*	Fossorial	n/a	24.57	Fibers	[58]
*Cerberus rynchops*	Mangrove	n/a	Confirmed	Polyester, PET fibers	[21]
*Hemidactylus* spp.	Insular terrestrial	n/a	Confirmed	Fibers, fragments	[74]

n/a, not available (sample size not reported in the original study).

## 7. Toxicological Effects of Microplastics in Reptiles

**Figure 3:**
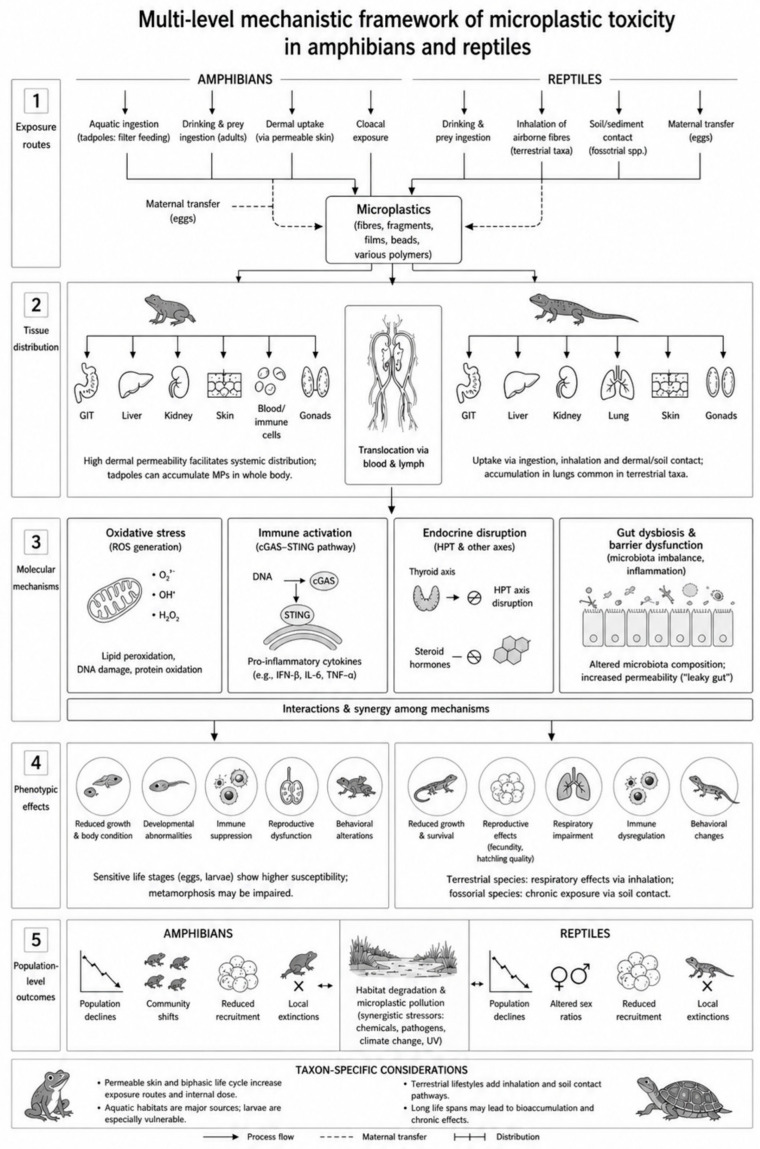
Multi-level mechanistic framework linking microplastic exposure to population-level outcomes in amphibians and reptiles. The framework is organised into five sequential levels: (1) exposure routes; (2) tissue distribution; (3) molecular mechanisms (oxidative stress, immune activation via the cGAS–STING pathway, endocrine/HPT-axis disruption, and gut dysbiosis/barrier dysfunction); (4) phenotypic effects; and (5) population-level outcomes, with taxon-specific considerations for amphibians and reptiles summarised at the foot of the figure. Amphibian and reptilian pathways are presented in parallel to highlight shared and divergent responses. Symbols: ✕, local extinction; ⊘, disruption or impairment of the indicated axis or process; ↔, interaction or synergy among mechanisms and stressors; ♀/♂, altered sex ratios. Arrow and line styles: solid arrow, process flow; dashed arrow, maternal transfer; ⊢⊣, tissue distribution.

Despite mounting evidence of widespread MP exposure across reptilian taxa, direct toxicological studies on reptiles remain substantially less developed than corresponding work on amphibians. The available evidence, while fragmentary, suggests that MP-induced pathological processes in reptiles share mechanistic parallels with amphibian responses while reflecting taxon-specific traits including cornified integument, prolonged gut transit times, and ectothermic metabolism (Figure 3).

### 7.1. Gastrointestinal and Systemic Effects

The gastrointestinal tract is the primary site of MP accumulation and toxicity in reptiles, as in amphibians, but the consequences of particle retention differ owing to the slower transit times characteristic of ectotherms with temperature-dependent digestive physiology. In sea turtles, both macro- and microplastic particles cause gastric impaction, false satiety reducing voluntary food intake below energetic maintenance, and intestinal obstruction, with even modest ingested volumes producing sublethal effects on growth, body condition, and reproductive output [1,64]. The systemic distribution of MPs across multiple organs in *Caretta caretta*, including cardiac and reproductive tissues, indicates that particle translocation from the gastrointestinal lumen to visceral organs is a feature of chronic exposure in long-lived reptiles with extended circulatory contact time [65].

In terrestrial squamates, the physical consequences of MP ingestion are compounded by the dietary ecology of carnivorous species, where prey items themselves carry MP body burdens that are transferred to the predator without opportunity for environmental dilution. For fossorial taxa such as *Blanus strauchi*, soil-contact exposure through ingestion of soil particles during fossorial locomotion and prey capture adds a quantitatively distinct exposure route absent in surface-active species [58]. Fibrous MPs ingested by snakes and lizards have been documented to accumulate in the intestinal lumen, where they may impair digestive enzyme access to food substrate and reduce assimilation efficiency, though direct mechanistic demonstration in reptiles awaits further experimental investigation.

### 7.2. Oxidative Stress and Inflammatory Responses

Available evidence from reptilian studies indicates that MP exposure activates oxidative stress pathways analogous to those documented in amphibians, and to those characterized for heavy metal exposure in reptiles, where ROS overproduction is buffered by metallothionein, glutathione, and antioxidant enzyme systems before chronic loads overwhelm compensatory capacity [12]. Upregulation of ROS, induction of antioxidant enzyme systems (SOD, catalase, glutathione peroxidase), and elevation of pro-inflammatory cytokines (TNF-α, IL-1β, IL-6) have been documented or inferred from ecotoxicological assessments in herpetofaunal taxa [21,59,60]. The chronic inflammatory state induced by persistent MP particle retention is consistent with the systemic organ distribution patterns observed in sea turtles, since inflammatory activation of vascular endothelium facilitates particle translocation from the gastrointestinal circulation to peripheral organ systems.

### 7.3. Reproductive and Developmental Toxicity

Maternal transfer of MPs to eggs in sea turtles, now documented in multiple species, represents a form of developmental toxicity that begins prior to embryonic life [65,66]. The embryotoxic consequences of in ovo MP exposure remain poorly characterized relative to larval amphibian exposure, but the co-occurrence of persistent organic pollutants, which adsorb preferentially to MP surfaces with MPs in sea turtle eggs, establishes a plausible mechanism for chemical developmental toxicity that merits experimental investigation. For freshwater reptiles experiencing MP exposure during critical reproductive periods, disruptions of yolk provisioning, egg incubation conditions, and hatchling energy reserves represent potential pathways through which MP contamination could reduce reproductive output, though direct experimental evidence in chelonians remains limited.

For non-oviparous reptilian species, reproductive toxicity through endocrine-disrupting MP additives presents an additional concern. Phthalates and BPA, documented in reptilian tissues from contaminated environments, exhibit antiandrogenic and estrogenic activity in non-avian reptile models, with potential to alter sex ratios, gonadal function, and reproductive behavior. Given that several reptile species exhibit environmental sex determination where incubation temperature dictates offspring sex, any MP-induced thermal perturbation of nest or incubation environments could produce skewed sex ratios with long-term population demographic consequences, independent of direct chemical endocrine disruption. This compounded threat parallels documented patterns of endocrine-disrupting contaminants combining with altered thermal regimes in TSD reptiles [12], suggesting that MP-borne endocrine-disrupting compounds and MP-altered nest microclimates could act through both pathways simultaneously.

## 8. Trophic Transfer and Ecosystem-Level Impacts

### 8.1. Microplastic Dynamics in Food Webs

Microplastics enter food webs through two complementary processes: direct environmental ingestion and trophic transfer, the latter referring to the consumption by predators of prey already bearing MP body burdens. Across aquatic ecosystems, MPs have been detected at multiple trophic levels from primary producers and zooplankton through invertebrates, fish, amphibians, and higher vertebrates, demonstrating pervasive bioaccumulation within organisms at each trophic step [78,79]. Although several field studies report positive associations between trophic level and MP burden, the current weight of evidence from systematic meta-analyses indicates that classical biomagnification, defined as a consistent increase in contaminant concentration per unit biomass with ascending trophic level, does not occur for MP particles under environmentally realistic conditions [78,79]. To avoid the apparent contradiction noted by readers of the earlier draft, it is essential to distinguish two separate phenomena throughout this section: (i) biomagnification of the plastic particles themselves, which the current meta-analytic evidence does not support; and (ii) biomagnification of the lipophilic chemical contaminants sorbed to or leached from those particles, which can follow conventional lipid-partitioning dynamics. These two processes are governed by different mechanisms—physical egestion and clearance for the particles, versus tissue partitioning for the associated chemicals—and should not be conflated (Table 4).

Several mechanistic considerations explain why classical biomagnification does not extend to MP particles in the way it does for lipid-soluble persistent organics. Active particle egestion at each trophic level substantially reduces particle load relative to dietary intake; gut retention time and digestive physiology vary widely across species, generating organism-specific clearance kinetics; and dilution effects emerge when small predators with high MP burdens are consumed by larger predators with substantially greater body mass. Critically, however, the chemical cargo carried by MPs, particularly lipophilic persistent organic pollutants, follows conventional biomagnification dynamics, because these compounds partition preferentially into lipid-rich tissues and are not subject to the same physical clearance mechanisms as the polymer particles themselves [7]. This chemical–physical dissociation means that trophic transfer of MPs delivers an accumulating chemical toxicant burden to higher-trophic-level organisms even where particle biomagnification does not occur [78].

**Figure 4:**
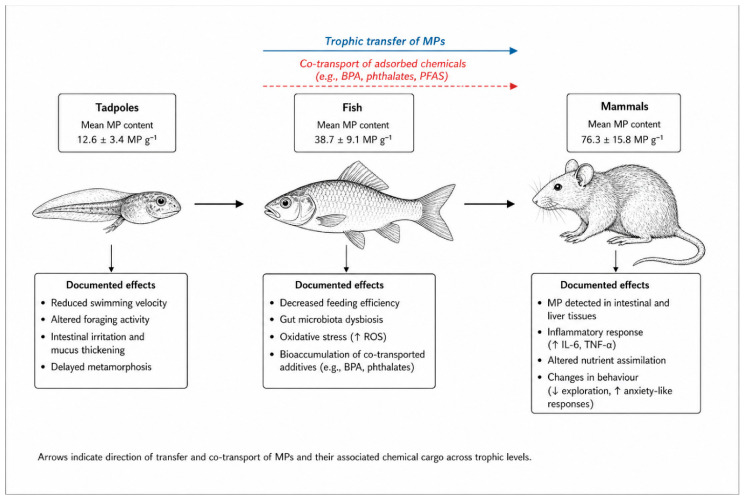
Trophic transfer pathways of microplastics across an amphibian–fish–mammal food chain. The schematic has been extended to include lower trophic levels (periphyton/biofilm and zooplankton) that supply microplastics to tadpoles, and to indicate that amphibians can also transfer microplastics to avian predators in addition to fish. Two arrow types now distinguish transfer of intact plastic particles from transfer of the sorbed/leached chemical cargo, which follow different accumulation dynamics (Section 8.1).

Amphibians and reptiles occupy critical positions in this trophic landscape as ecological bridges between aquatic and terrestrial food webs. Amphibian larvae ingest MPs from water, sediment, and contaminated prey, and particles may persist through metamorphosis in tissues or be transferred to predators during larval predation. Tadpoles thus function as early-stage trophic vectors, facilitating MP movement to adult amphibians and to higher consumers, including wading birds, snakes, and small mammals [26,29]. Direct experimental quantification was achieved in a controlled study demonstrating measurable MP transmission from *Physalaemus cuvieri* tadpoles to fish and subsequently to mammalian receivers, with MPs detectable at all three trophic levels and behavioral impairments, reduced locomotion, and elevated anxiety indices documented in mammalian recipients [39]. The Axolotl (*Ambystoma mexicanum*) has likewise been shown to accumulate MPs through zooplankton prey, with impaired predation efficiency at environmentally relevant exposure concentrations [80] (Figure 4).

### 8.2. Ecosystem-Level Consequences

The ecological significance of MP trophic transfer extends beyond individual organism toxicity to encompass disruption of food web structure, nutrient cycling, and ecosystem service provision. MP-induced behavioral impairments in amphibian prey alter trophic interaction rates and shift predator–prey encounter probabilities, potentially destabilizing the regulatory function that amphibians perform as mid-trophic consumers controlling invertebrate prey populations. At the ecosystem level, MP-induced immunosuppression operating through gut microbiome disruption and the Trojan horse co-contaminant mechanism facilitates pathogen amplification within and across trophic levels [56,81,82], and the hypothesized synergy between MP contamination and *Batrachochytrium dendrobatidis* represents a particularly consequential dynamic given the central role of amphibians in biological pest control, periphyton grazing, and energy subsidy to terrestrial predators [21,44].

## 9. Anthropogenic Pressure and Global Geographic Patterns

### 9.1. The Urban–Rural Gradient in Exposure

A robust positive association between anthropogenic activity indices and microplastic burdens in herpetofaunal tissues has been documented consistently across multiple continents, providing strong correlational evidence that urbanization, industrial land use, and inadequate waste management infrastructure are the primary proximal drivers of MP exposure in amphibians and reptiles. Across European, Asian, and American study systems, herpetofauna in urban and peri-urban habitats consistently exhibit higher individual MP burdens, greater morphological diversity of ingested particles, and broader polymer-type spectra than conspecifics from rural or remote environments.

**Figure 5:**
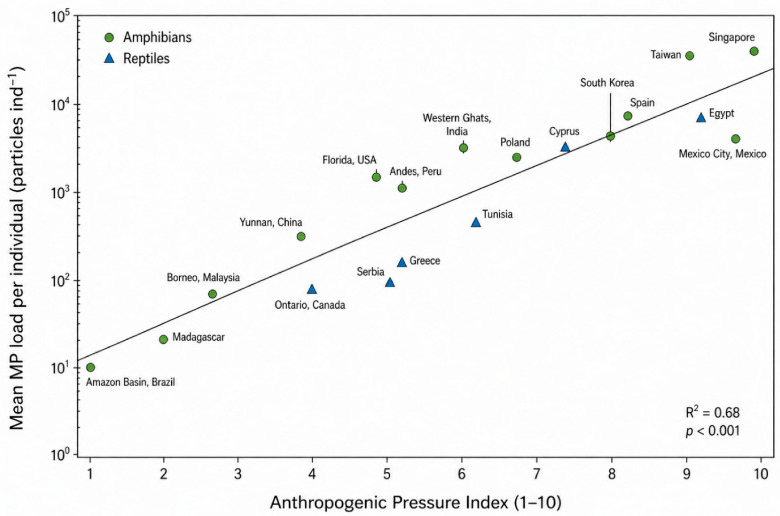
Relationship between catchment-level anthropogenic pressure and mean microplastic load in amphibians and reptiles, compiled from published comparative studies.

The spatial pattern is particularly well characterized in Türkiye, where populations of *Pelophylax* species in metropolitan Istanbul catchments carry substantially higher MP loads than populations from remote mountain lakes such as Şavşat Karagöl [24,41]. This urban-to-rural gradient, quantified by Levin’s niche breadth index, suggests that anthropogenic pressure shapes not only the abundance but also the compositional diversity of MPs available for amphibian ingestion. Comparable analyses across multiple European amphibian species have demonstrated that increases in catchment-level anthropogenic pressure correspond to statistically significant increases in both MP load and morphological diversity in larvae [25], a dose–response relationship with important implications for ecological risk assessment in urbanizing catchments (Figure 5).

The urban–rural gradient does not imply that rural or protected habitats are contamination-free. As documented across endemic *Neurergus* populations in remote Turkish spring habitats and *Pelophylax* populations in Turkish conservation lakes, atmospheric long-range transport delivers MPs to geographically isolated populations [34,41]. The gradient therefore describes relative, not absolute, contamination status, and the absence of zero-contamination reference sites in many regions complicates the establishment of pristine baselines for ecological risk threshold derivation.

### 9.2. Land Use, Industrial Activity, and the Trojan Horse in Agricultural Systems

The composition and concentration of MPs in environmental matrices are strongly shaped by the dominant land use pattern within a catchment. Industrial discharge particularly from textile manufacturing, which releases high concentrations of synthetic fiber MPs, constitutes a point-source input that produces elevated fiber contamination in downstream receiving waterbodies, with corresponding impacts on amphibian and reptile populations. Agricultural zones contribute MP pollution through diffuse pathways: polyethylene mulch films, irrigation pipes, and greenhouse covers fragment into soil MPs that migrate into adjacent waterbodies through erosion and runoff. These agricultural-origin MPs carry agricultural chemical sorption loads including malathion, carbendazim, and other pesticide residues that compound the toxicological burden for organisms contacting these particles through the Trojan horse mechanism [80,83].

The relationship between artificial land cover fraction and MP burden in amphibian larvae has been quantified in multiple studies [25,36], establishing land use indices as predictive variables for MP exposure risk in herpetofaunal communities.

### 9.3. Protected Areas and the Limits of Spatial Conservation

Conservation-designated protected areas are increasingly demonstrated to be insufficient barriers against MP contamination of herpetofaunal populations. This insufficiency stems from two structural limitations. First, strictly protected areas cover only 3.4% of the global amphibian distribution range and 3.5% of the global reptile distribution range, among the lowest coverage values for any vertebrate class and substantially below comparable figures for birds and mammals [11,17]. This fundamental representational gap means that the majority of herpetofaunal populations reside outside any formal protection, regardless of MP considerations. Second, and more fundamentally, MP pollution is not constrained by protected area boundaries: atmospheric deposition, hydrological connectivity, and faunal movement across boundaries all facilitate MP infiltration into formally protected landscapes.

Empirical evidence from protected ecosystems confirms this boundary permeability. Amphibian populations in Borçka Karagöl conservation lake (Türkiye), Komchén de los Pájaros wildlife reserve (Mexico), and conservation areas in Southeast Asia all accumulate MPs attributable to atmospheric deposition, tourist activities within reserves, and transboundary aquatic transport [26,84]. These findings highlight a structural inadequacy in current conservation policy: protected zones define spatial boundaries for human activity but cannot exclude the long-range transport mechanisms that deliver MPs from source regions to ecologically sensitive receptor habitats. Effective MP mitigation for herpetofauna therefore requires not only protected area expansion but source-reduction interventions operating at the catchment and atmospheric scales.

### 9.4. Regional and Continental Disparities in Research and Risk

Geographic analysis of the MP research literature on herpetofauna reveals a pronounced concentration in the Global North, particularly Europe, the eastern United States, and East Asia, while the regions harboring the greatest amphibian and reptile diversity remain substantially under-sampled. Amphibian diversity is concentrated in the Neotropics, Sub-Saharan Africa, and Southeast Asia; reptile diversity peaks in the tropics across all three continental landmasses. These regions coincide with areas of rapid plastic pollution growth, limited waste management infrastructure, and insufficient research investment, generating a tripartite compounding of biodiversity value, contamination risk, and knowledge deficit. Within the Mediterranean basin, Anatolian-Turkish research groups have produced a particularly dense empirical literature on amphibian and reptile MP exposure spanning multiple species, life stages, and habitat types—a regional density that reflects sustained investigative effort rather than a true global gradient and therefore requires careful framing when extrapolating to other Mediterranean or Middle Eastern systems where comparable data have not yet been generated.

Regional contamination data illustrate the scale of exposure heterogeneity. MP ingestion rates in studied populations range from 8.3% in Turkish terrestrial lizards in rural habitats to near-universal detection in sea turtles from major ocean basins and in some highly contaminated freshwater systems. The Amazon River, draining one of the world’s most biodiverse herpetofaunal regions, discharges an estimated 3.22 × 10^5^ tons of plastic waste annually, and recent studies confirm pervasive MP contamination across water, sediment, and biota in Amazonian systems [37]. The Mediterranean basin functions as a global plastic accumulation hotspot, with MP concentrations in coastal waters substantially exceeding those of major oceanic gyres. Even the Arctic, historically considered a pristine reference environment, now carries measurable MP loads attributable to long-range atmospheric and oceanic transport, demonstrating the global reach of MP pollution that erases geographic distinctions between contaminated and reference conditions [5].

## 10. Knowledge Gaps and Future Research Priorities

The following research priorities are presented as hypothesis-driven directions that, if addressed systematically, would advance the field from its current descriptive phase toward quantitative ecological risk assessment for herpetofauna.

### 10.1. Priority 1 (Immediate): Methodological Standardization

**Hypothesis** **1.** *Adoption of a standardized minimum reporting framework encompassing particle size range (µm Ferret diameter), morphological classification (ISO 24187:2023-compliant) [85], polymer type confirmation (ATR-FTIR or Raman spectroscopy), tissue of analysis, and contamination blank QA/QC protocols would reduce inter-study variability by >50% and enable the first globally valid meta-analysis of MP effects in herpetofauna.*

Currently, inconsistent reporting units (particle count versus mass versus volume), variable size thresholds, and differing spectroscopic protocols prevent meaningful cross-study comparison and obscure genuine biological signals in exposure data [21,86]. A particular methodological need is the development of species-specific MP extraction protocols adapted to the high mucus content of amphibian skin, the keratinized integument of reptiles, and the organic-enriched matrices of freshwater sediments, all of which interfere with existing digestion protocols optimized for fish tissue or marine sediment [83,87].

### 10.2. Priority 2 (Immediate): Filling the Reptile Toxicology Gap

**Hypothesis** **2.** *Reptilian physiological characteristics, including cornified integument with low permeability, longer gut retention times, and lower metabolic rates relative to endotherms, produce distinct bioaccumulation kinetics and tissue distribution patterns for MPs compared to amphibians.*

Testing this hypothesis requires comparative toxicokinetic studies conducted across reptilian orders under standardized experimental conditions. The complete absence of toxicological data for Crocodilian (14 extant species, several critically endangered), Tuatara (*Sphenodon punctatus*, New Zealand’s sole *Rhynchocephalia* representative), and the majority of snake and lizard families represents gaps that should be prioritized given the evolutionary distinctiveness and conservation vulnerability of these lineages. As a research priority, the development of non-lethal tissue sampling protocols using shed skin, feces, and oral swabs would enable repeated sampling of threatened species without additional mortality pressure.

### 10.3. Priority 3 (Short-Term): Transgenerational and Epigenetic Effects

**Hypothesis** **3.** *Maternal transfer of MPs to eggs, demonstrated in Caretta caretta and mechanistically plausible in other oviparous herpetofauna, induces epigenetic modifications (DNA methylation patterns, histone acetylation states) in embryonic tissues that alter developmental gene expression programs and produce heritable legacy effects in offspring phenotype, independent of direct offspring MP exposure.*

This hypothesis is testable through multi-generational mesocosm experiments in model amphibian species (*Xenopus laevis*, *Rana temporaria*) coupled with whole-genome bisulfite sequencing (WGBS) to characterize methylation landscapes in F1 and F2 generations relative to MP-naïve F0 controls. The detection of MP particles and associated chemical cargo in amphibian embryos would first require demonstration analogous to that established for sea turtles, constituting a foundational empirical priority for this research direction.

### 10.4. Priority 4 (Short- to Medium-Term): Mechanistic Basis of MP–Bd Synergy and Translation into Population Viability

**Hypothesis** **4.** *MP-induced dysbiosis of the cutaneous microbiome specifically reduces the abundance of Bd-antagonistic bacterial taxa (particularly Janthinobacterium lividum and Lysobacter gummosus, producers of Bd-inhibitory violacein and lytic enzymes) and suppresses production of antimicrobial peptides (AMPs) by skin glands, creating a mechanistic pathway from plastic pollution to increased chytridiomycosis severity that is independent of, and additive to, general systemic immunosuppression.*

This hypothesis is directly testable through fully factorial experiments (MP exposure × Bd challenge × cutaneous microbiome characterization by 16S rRNA metagenomic profiling × AMP quantification by mass spectrometry) in a susceptible model species, ideally one with well-characterized natural Bd dynamics, such as *Rana temporaria* or *Alytes obstetricans*.

The conservation translation of these mechanistic findings is the integration of empirically measured MP exposure effects, such as reduced larval survival, delayed metamorphosis, increased pathogen susceptibility, and compromised juvenile fitness, as stressor parameters within population viability analysis (PVA) models for critically endangered amphibians, with MP-amplified Bd dynamics representing the most plausible single pathway through which contemporary MP pollution could measurably accelerate extinction risk in already-imperiled populations. Achieving this integration requires close collaboration between ecotoxicologists generating effect-parameter estimates and conservation biologists with species-specific demographic models, and would directly support the case for incorporating MP burden into IUCN Red List threat assessments (Section 11).

### 10.5. Priority 5 (Long-Term): Thermal Physiology and Climate Change Interaction

**Hypothesis** **5.** *The thermal window for MP-mediated endocrine disruption, particularly HPT axis interference in ectothermic herpetofauna, narrows under climate warming scenarios, such that species already operating near their thermal tolerance limits experience MP-induced developmental disruption at lower MP concentrations than populations in thermally stable environments.*

This interaction would mean that the effective ecotoxicological risk of MP contamination is not static but increases with ambient temperature, a dynamic with profound implications for conservation planning in the context of concurrent MP pollution and climate warming. Comparable temperature × pollutant interactions have been highlighted for amphibian and reptile detoxification physiology, where elevated ambient temperatures impair enzymatic detoxification capacity and amplify the toxicity of co-occurring chemical stressors [12], providing a mechanistic basis for predicting analogous synergies in MP–climate experiments. Multi-factor experimental designs combining temperature gradients (+0 °C, +1.5 °C, +3 °C above ambient) with realistic MP mixture exposures and measuring thyroid hormone profiles, metamorphic timing, thermal tolerance indices, and survival would directly test this hypothesis in model amphibian species.

### 10.6. Priority 6 (Long-Term): Evolutionary and Population-Genetic Consequences of Microplastic Exposure

**Hypothesis** **6.** *Chronic, geographically structured MP exposure imposes measurable selective pressure on amphibian and reptile populations by reducing fitness in individuals with traits that elevate exposure (e.g., suspension-feeding intensity in larvae, dietary breadth, microhabitat use, integument permeability) and by amplifying drift through population-level demographic decline, with detectable consequences for genetic diversity, effective population size, and adaptive trait variance in MP-contaminated catchments relative to comparable reference populations.*

Three complementary research designs would test this hypothesis directly. First, paired population-genetic comparisons across MP-contamination gradients using reduced-representation sequencing (RAD-seq, ddRAD) or whole-genome resequencing in amphibian and reptile populations occupying paired urban–rural catchments with quantified MP burdens could detect signatures of MP-associated selection (Fst outlier scans, environmental association analyses) and quantify reductions in effective population size in heavily contaminated populations. Second, common-garden experiments across populations from contrasting MP-exposure regimes would test whether observed fitness-relevant phenotypes (growth rate, behavioral tendency, immune competence) are heritable and have shifted in directions predicted by the differential-exposure hypothesis. Third, longitudinal monitoring of trait distributions and microsatellite/SNP-based diversity indices in populations sampled across the recent decades of accelerating MP accumulation could provide direct contemporary-evolution evidence, particularly when archived specimen collections enable historical baselines. Establishing these baselines in heavily impacted regions, including the Anatolian and Mediterranean systems where ecological MP burden has been most thoroughly characterized, represents an immediate priority for evolutionary conservation biology.

Emerging priority—biodegradable and bio-based plastics. As biodegradable and bio-based polymers (for example, polylactic acid, PLA, and polyhydroxyalkanoates, PHA) are increasingly promoted as replacements for conventional petroleum-derived plastics, a pressing open question is whether these materials reduce, leave unchanged, or merely alter the hazard they pose to amphibians and reptiles. Exposure of Pelophylax nigromaculatus tadpoles to PLA-MPs at environmentally relevant concentrations negatively affected survival, growth, and development, and reduced locomotor performance in open-field tests [88], indicating that ‘green’ biopolymers are not toxicologically inert in anuran larvae. Because biodegradable polymers can also fragment more rapidly, sorb pesticides at higher rates, and shift gut-microbiome composition, future research priorities should explicitly include comparative toxicity testing of biodegradable versus conventional MPs across amphibian and reptilian models, so that the adoption of new materials does not inadvertently exchange one ecotoxicological hazard for another.

## 11. Ecological Risk Characterization, Conservation, and Policy Implications

Realizing meaningful reduction in microplastic exposure for amphibians and reptiles requires the integration of source-reduction policy, ecological engineering of habitat quality, species-specific monitoring, and modification of international conservation frameworks to formally recognize MP pollution as a quantifiable extinction threat.

### 11.1. Source Reduction and Policy

Urban runoff and wastewater effluent represent the primary pathways through which MPs from consumer and industrial sources enter freshwater ecosystems. Upgrading sewage treatment infrastructure with tertiary filtration technologies capable of retaining particles in the 10–500 µm range—the size classes most frequently documented in amphibian tissues—is a technically feasible and demonstrably effective intervention [37]. Complementary policy measures targeting upstream source-reduction restrictions on single-use plastics, mandatory biodegradability standards for agricultural mulch films, and extended producer responsibility frameworks for synthetic textile manufacturers address the production of primary and secondary MPs before they enter the environment. At the international level, the proposed Global Plastics Treaty represents a mechanism for coordinating national plastic reduction commitments, and its implementation should explicitly include provisions for the protection of freshwater and terrestrial biodiversity hotspots where herpetofaunal vulnerability is greatest.

### 11.2. Habitat Management and Monitoring

Riparian buffer zone restoration reduces MP transport from terrestrial to aquatic systems by providing physical filtration and reducing surface runoff velocity. For amphibian breeding habitats, maintaining or restoring intact riparian vegetation along watercourse margins constitutes a cost-effective, multi-benefit intervention that simultaneously reduces MP loading, improves water quality, provides thermal buffering of larval habitats, and enhances habitat connectivity for adult amphibian movement. Monitoring programs must be redesigned to prioritize high-pressure catchments where MP burdens in herpetofauna consistently exceed ecological risk thresholds, while simultaneously ensuring that protocols are sufficiently standardized to enable cross-site and cross-taxa comparison [26].

### 11.3. Integration into Conservation Frameworks

The most consequential policy recommendation emerging from this review is the case for future consideration of MP burden as a candidate stressor criterion in IUCN Red List threat assessments, national biodiversity action plans, and environmental impact assessment frameworks. We emphasize that such incorporation would be premature until a robust, quantitative link between MP burden and population-level demographic outcomes has been established; the present recommendation is therefore framed as a research-and-policy direction rather than a call for immediate reclassification. Current IUCN threat categories recognize pollution among primary threats, but the operational classification is insufficient to capture the mechanistically distinct hazard posed by MP contamination; developing standardized MP exposure thresholds derived from laboratory effect data calibrated to field-realistic concentrations that trigger elevated threat classification would provide actionable conservation guidance for amphibians and reptiles [45]. Citizen science programs leveraging existing amphibian population monitoring networks could substantially expand spatial and taxonomic coverage of exposure data.

### 11.4. Toward Herpetofauna-Specific Ecological Risk Assessment

The operational translation of the evidence reviewed here into a quantitative ecological risk characterization (ERC) framework for herpetofauna remains constrained by three interlocking structural deficits. First, the chemical and morphological heterogeneity of environmentally encountered MP burdens—variable polymer composition, particle size distributions, fiber versus fragment versus film morphology, weathering state, and co-contaminant load—defies the single-substance dose–response paradigm that underpins conventional ERC procedures. Second, the laboratory toxicity database for amphibians is dominated by a small number of model species (*Xenopus laevis*, several Rana taxa, *Physalaemus cuvieri*) tested under acute exposure regimes at supra-environmental concentrations [9,43,47,48,52], while for reptiles, it remains restricted almost entirely to descriptive ingestion surveys without controlled toxicokinetic measurements [21,58,70]. Third, the species sensitivity distributions (SSDs) that ecological risk assessment frameworks routinely employ to derive predicted no-effect concentrations cannot yet be constructed for herpetofauna because chronic, partial-life-cycle, environmentally realistic exposure data exist for too few species and too few endpoints to support robust statistical inference.

A pragmatic intermediate step toward herpetofauna-specific ERC is agreement on a minimum endpoint battery that future toxicity studies should prioritize. Drawing on the mechanisms documented in Section 5 and Section 7, four endpoint categories meet the criteria of mechanistic specificity, ecological relevance, and methodological tractability: (i) developmental endpoints relevant to amphibian metamorphic competence (T3/T4-dependent timing, hindlimb morphometry, post-metamorphic survival); (ii) immune–disease endpoints integrating cutaneous microbiome composition (16S rRNA profiling), antimicrobial peptide quantification, and standardized pathogen challenge response (Bd zoospore equivalents, ranavirus FV3 titer); (iii) histopathology endpoints in primary target organs (intestinal epithelial integrity, hepatic parenchymal architecture, gonadal differentiation); and (iv) behavioral endpoints reflecting fitness-relevant locomotor, anti-predator, and feeding performance. Adoption of such a battery within standardized exposure protocols using field-realistic MP mixtures rather than pristine virgin particles would generate the comparable, multi-species effect data needed to derive provisional protective threshold concentrations and ultimately to construct herpetofauna-specific SSDs.

Realistic ERC for herpetofauna must additionally accommodate the chemical mixture problem inherent to MP exposure. The Trojan horse co-contaminant transport mechanism (Section 3.2), the leaching of plasticizer, flame-retardant, and stabilizer additives from the polymer matrix under physiological conditions, and the demonstrated co-occurrence of MP pollution with agrochemical, heavy metal, and pharmaceutical residues in the freshwater habitats most commonly occupied by amphibians [80,83] together imply that the toxicologically relevant exposure unit is not the isolated polymer particle but the particle–additive–sorbed-contaminant complex. Concentration addition and independent action models routinely applied to chemical mixtures in regulatory ecotoxicology can, in principle, be extended to MP-associated mixtures, but their parameterization for herpetofauna requires component-specific effect data that are largely unavailable. Until such data accumulate, an interim risk-based approach combining MP burden quantification with a screening-level estimate of associated chemical cargo (e.g., per-particle phthalate, BPA, and PAH equivalents) offers a tractable bridge between the current descriptive evidence base and the regulatory toxicology frameworks within which conservation policy decisions are ultimately made [45].

## 12. Conclusions and Future Directions

This review integrates global evidence on the exposure, bioaccumulation, and toxicological consequences of microplastic pollution in amphibians and reptiles, two vertebrate classes that, despite representing approximately 20% of global tetrapod diversity and performing irreplaceable ecological functions, remain substantially underrepresented in the ecotoxicological literature relative to fish, birds, and mammals. The contamination landscape is geographically pervasive and taxonomically non-selective: MPs have now been documented from Amazonian rainforest streams, high-altitude montane springs in Türkiye, remote Pacific nesting beaches, and biodiverse freshwater systems across five continents, with prevalence ranging from 8.3% in terrestrial lizards in rural Mediterranean habitats to near-universal detection in marine turtle populations and tadpole communities from high-anthropogenic-pressure catchments. The dominant ingested forms—fibrous PET and PP particles—are consistent across taxa, regions, and habitat types, implicating global synthetic textile and packaging industries as the dominant source categories driving herpetofaunal exposure.

The toxicological consequences of this exposure operate across multiple levels of biological organization with demonstrable ecological consequences. At the molecular level, MPs activate oxidative stress cascades through ROS overproduction; disrupt HPT axis function, impairing metamorphic regulation; and induce gut microbiome dysbiosis, weakening mucosal immune defenses. An additional pathway involving innate immune activation via cGAS/STING DNA-sensing has been characterized in mammalian models [20] and remains to be tested directly in amphibian and reptilian systems. At the organismal level, these molecular insults manifest as growth impairment, developmental delay or acceleration, locomotor and anti-predator behavioral deficits, hepatic and gastrointestinal histopathology, reproductive toxicity, and critically enhanced susceptibility to infectious pathogens. The potential synergistic interaction between MP-induced immunosuppression and *Batrachochytrium dendrobatidis* is of particular conservation significance: chytridiomycosis has already driven at least 90 amphibian species to extinction and caused population declines in at least 500 more, and any pollutant interaction that amplifies disease severity in an already-devastated taxon demands urgent scientific and policy attention.

Three structural asymmetries define the current state of the field and constrain global risk assessment: a taxonomic bias concentrating research on anurans and sea turtles while leaving salamanders, caecilians, freshwater turtles, terrestrial lizards, snakes, and crocodilians without substantive characterization; a geographic bias localizing studies in the Global North and Mediterranean basin while regions hosting the greatest herpetofaunal biodiversity remain severely under-sampled; and an experimental bias toward acute, supra-environmental laboratory exposures using pristine polymer particles, producing toxicity estimates of uncertain relevance to chronic, weathered field mixtures. Advancing from descriptive contamination surveys toward quantitative ecological risk assessment requires standardized, spectroscopically confirmed MP characterization enabling cross-study meta-analysis; comparative toxicokinetic studies across underrepresented reptilian orders; multi-generational experiments on transgenerational and epigenetic effects; mechanistic characterization of the MP–microbiome–Bd interaction translated into population viability models; multi-stressor experiments combining realistic MP exposures with climate warming scenarios; and population-genetic and common-garden tests of contemporary MP-driven selection on fitness-relevant traits.

From a regulatory toxicology and conservation policy perspective, this review proposes that MP burden be evaluated as a candidate stressor criterion for possible future incorporation into IUCN Red List threat assessments, national biodiversity monitoring programs, and watershed environmental impact frameworks, and that herpetofauna-specific ecological risk characterization be developed through a coordinated program of standardized, mechanism-resolved, environmentally realistic toxicity testing capable of supporting species sensitivity distributions and protective threshold derivation. Amphibians and reptiles, as ectotherms with intimate dependencies on environmental quality, function as ecological sentinels of the broader consequences of plastic pollution. Their documented impairment by MP exposure, from molecular disruption of thyroid hormone signaling in developing tadpoles to maternal transfer of plastic particles to sea turtle embryos, reflects broader consequences of inadequate plastic management that extend beyond these taxa. Addressing this crisis will require coordinated effort spanning materials science, regulatory ecotoxicology, conservation biology, and environmental policy.

## Figures and Tables

**Table 1:** Summary of microplastic contamination studies in amphibians across geographic regions. Contamination rates represent individual-level detection prevalence where available. Because the compiled studies differ in analytical approach, readers should note that identification methods range from stereomicroscopy alone to ATR-FTIR-, Raman-, or pyrolysis-GC/MS-confirmed polymer typing, and that reported lower size thresholds vary (commonly ≈ 10–100 µm); these methodological differences limit strict quantitative comparison across rows.

Species/Group	Country	*n*	Contamination (%)	Dominant MP Type	Reference
*Pelophylax* spp. (tadpoles)	Türkiye	276	73–80	Fibers (57–62%), PET	[24]
*Hyla savignyi*	Türkiye	200	56.5	Fibers (76%), PET	[42]
*Hyla orientalis*	Türkiye	76	11.8	Fibers, PET	[42]
*Ommatotriton* spp.	Türkiye	91	29–43	Fibers, PET	[33]
*Neurergus* spp.	Türkiye	35	60–70	Fibers, PET	[34]
*Alytes obstetricans*	Spain	48	87.5	PE microspheres	[43]
*Physalaemus cuvieri*	Brazil	120	100	PE particles	[39]
*Amietia delalandii*	S. Africa	n/a	Confirmed	Fibers	[31]
*Rana sylvatica*	Canada	Mesocosm	Exp.	MP mixture (field-relevant)	[44]
Multi-species tadpoles	Poland	201	26	Fibers (97%)	[23]
*Rana amurensis*	Russia (Siberia)	18	83 (adults)	Fibers (84.6%)	[32]

n/a, not available (sample size not reported in the original study).

**Table 2:** Selected sublethal effects of microplastics documented in amphibians, grouped by effect category, focal species, and exposure conditions. Concentrations are reported in the units used by the original studies; where these differ (particles mL^−1^, mg L^−1^, µg mL^−1^), they are not directly interconvertible and are retained verbatim to avoid implying a precision the primary data do not support.

Effect Category	Species	MP Type/Concentration	Observed Effects	Reference
Growth and development	*Alytes obstetricans*	PE, 10^3^ particles mL^−1^	Reduced feeding, growth retardation, elevated mortality	[43]
Metamorphic delay	*Rana sylvatica*	MP mixture, mesocosm	Delayed metamorphosis, reduced post-metamorphic survival	[44]
Metamorphic acceleration	*Xenopus laevis*	PE-MPs, environmental	Elevated metabolic rate, altered juvenile morphology	[48]
Behavioral	*Physalaemus cuvieri*	PE, 60 mg L^−1^	Reduced locomotion, weakened anti-predator response	[39]
Gastrointestinal	*Xenopus laevis*	Polyester fibers, 6.3 µg mL^−1^	Epithelial damage, luminal obstruction	[51]
Cytotoxicity	*Physalaemus cuvieri*	PE, 60 mg L^−1^	Erythrocyte mutagenicity, morphological abnormality	[52]
Hepatic injury	Mouse (mammalian model)	0.1 µm MPs	DNA damage, cGAS/STING activation, fibrosis	[20] (mammalian; mechanism inferred for amphibians)
Disease susceptibility	*Alytes obstetricans*	PE microspheres	Dose-dependent increase in Bd load	[57]
Endocrine (HPT)	*Xenopus laevis*	PS-MPs + additives	Thyroid hyperplasia, disrupted T3/T4 regulation	[54]
Oxidative stress	Several tadpole taxa	PE, PS, PET	Elevated SOD, catalase, corticosterone	[50]

**Table 4:** Documented trophic transfer events involving microplastics across food chains relevant to amphibians and reptiles. The type of evidence (field versus experimental versus review) is indicated in the Study column; a purely marine copepod–jellyfish chain included in the original draft has been removed because it does not involve amphibian or reptilian receivers. Where reported, the transferred polymer is polyethylene (rows 1–2); the Turkish entry reflects an inferred habitat–organism correlation rather than a controlled transfer experiment.

Study	Trophic Chain	Transfer Confirmed	Effect in Receiver	Reference
Brazil (experimental)	*P. cuvieri* tadpoles → fish → mammal	Yes	Reduced locomotion, anxiety in mammals	[39]
Mexico (experimental)	Zooplankton → Axolotl (*A. mexicanum*)	Yes	Impaired predation efficiency	[80]
Türkiye (field)	Environment → tadpoles → adult *Pelophylax* spp.	Inferred	Habitat–organism MP profile correlation	[26]

## Data Availability

No new data were created or analyzed in this study. Data sharing is not applicable to this article.
